# Managers' Practices of Tobacco and Marijuana Smoking Policies in Hispanic-Occupied Multiunit Housing

**DOI:** 10.1089/heq.2018.0100

**Published:** 2019-07-03

**Authors:** Angelica Delgado Rendon, Tess Boley Cruz, Lourdes Baezconde-Garbanati, Claradina Soto, Jennifer B. Unger

**Affiliations:** Department of Preventive Medicine, Keck School of Medicine, Institute for Prevention Research, University of Southern California, Los Angeles, California.

**Keywords:** secondhand smoke, manager, multiunit housing, tobacco, marijuana, e-cigarettes, cannabis

## Abstract

**Purpose:** This study assessed the knowledge, attitudes, and practices of managers of Hispanic-occupied multiunit housing (MUH) related to the prevalence and prevention of secondhand smoke (SHS), thirdhand tobacco smoke, and secondhand marijuana smoke (SHMS).

**Methods:** A narrative analysis was conducted of 20 interviews with live-in apartment managers. Their opinions on policies and an educational fotonovela were also gathered.

**Results:** The properties were located in 10 cities within the Los Angeles County, representing a wide array of local policies and practices. Only two managers were correctly informed of the existing MUH smoking policies in their cities. Participants reported ambiguity in city laws and company rules regarding smoking. Managers do not distinguish between smoking recreational marijuana and medicinal marijuana. Several respondents believed the landlords have more power to create rules. Most favored a total ban on smoking of all substances on the premises.

**Conclusions:** Most managers report low agency in being able to pass no-smoking rules. Participants support smoking policies that include all smokable products. Managers would like new government policies, manager trainings, tenant education, and ways to enforce rules to protect apartment tenants from SHS and SHMS. Educational interventions should coincide with the timing of key manager/tenant activities. Results can be used in policy development and educational interventions.

## Background

Hispanic multiunit housing (MUH) dwellers are disproportionally affected by the effects of tobacco secondhand smoke (SHS) and thirdhand smoke (THS). The tobacco smoking prevalence among U.S. adults has decreased over the last 25 years largely due to the proliferation of clean air policies in public spaces.^1,2^ Apartment tenants where no-smoking policies have been implemented do report a decrease in exposure to involuntary smoke from 31% to 23.6% at postmeasurements.^3^ Notwithstanding increases in household and public housing smoking bans, smoke infiltrates from neighboring units, sidewalks, and even from surrounding buildings. Therefore, present policy measures only partially address exposure to these toxicants in public and shared places.^4^

Despite more favorable attitudes for no-smoking policies, exposure to environmental smoke for low-income Hispanic apartment tenants remains unavoidable.^5–7^ Low-income Hispanic apartment residents are at high risk of exposure.^8^

For some inner-city Hispanic dwellers, language and cultural factors are barriers to take action against this exposure.^9–11^ A needs assessment conducted with residents of apartment buildings in Los Angeles County revealed that many Hispanic residents continue to be exposed to involuntary tobacco smoking despite having no-smoking tobacco policies in rental contracts^6^ and may be exposed to smoke from medicinal and recreational marijuana (secondhand marijuana smoke [SHMS]). Recent studies have shown that the indoor use of electronic cigarettes generates similar air pollution levels to cigarette SHS, thus contradicting the belief that vaping is a healthy alternative to regular tobacco.^12–14^

Marijuana smoke carries some of the same carcinogens as tobacco smoke.^15^ Marijuana is a psychotropic drug with symptom relieving properties for multiple health conditions such as anorexia, cancer, epilepsy, muscular degenerative conditions, and chronic pain.^16^ With the recent approval of the Medicinal and Adult-Use Marijuana Regulation and Safety Act in California, which regulates the commercial medicinal and recreational use of marijuana, the timeliness of this analysis helps us understand the etiology of the evolving smoke pollution problem.^17^

The long-term effects of SHS of tobacco on people's health are well documented.^18^ These effects are especially damaging to people with chronic conditions, such as asthma, lung diseases, and cancer. Marijuana smoke contains more carcinogens than tobacco smoke.^19^ While the long-term effects of SHMS have not been thoroughly evaluated, persistent firsthand use of marijuana has been documented to cause cognitive impairment, respiratory illnesses, fertility problems, and increased risk of certain types of cancer.^19,20^

MUH managers are key to on-site prevention efforts of environmental exposure to SHS, THS, and SHMS; however, it is not known whether they are able to create and enforce smoking rules. In 2016, the Department of Housing and Urban Development (HUD) passed a smoke-free rule for all public housing.^21,22^ This rule restricted the use of any tobacco products within living areas, outdoor common areas, and 25 feet around the building. Marijuana is a prohibited substance at the federal level and therefore not allowed in HUD-funded properties already. Marijuana is allowed in non-HUD buildings in California that do not have smoke-free rules. It is not known if the managers are able to enforce federal and local laws against smoking in apartment properties and if not, what barriers and facilitators exist.

This is a qualitative study stemming from a larger intervention to assess the effect of a low literacy graphic novel, *El Reto de Marta* (*Marta on a Mission)*, to educate Hispanic apartment tenants about the dangers of involuntary smoke pollution in Los Angeles County.^6^ The purpose of this study was to survey on-site managers of apartment buildings to advance our understanding of the prevalence of SHS, SHMS, and THS, and explore their educational and policy preferences.

The research questions were as follows. (1) What is the managers' knowledge of SHS/SHMS/THS? (2) What are the housing companies' rules about smoking tobacco and marijuana? (3) What policies do the managers prefer? (4) What are the benefits and barriers to smoke-free buildings? (5) Would the managers find an educational fotonovela, *Marta on a Mission*, helpful to educate tenants about SHS/SHMS?

## Methods

### Data collection

Potential participants were identified using a four-step process described in a previous publication.^6^ For the purposes of this study, MUH was defined as a housing structure containing at least 10 units in geographic areas of Los Angeles County with a high concentration of Hispanics. The research staff inquired about whether there was a manager on-site. Only one adult per MUH could participate. The eligibility criteria were that participants be adults 18 years or older, current live-in apartment manager, speak English or Spanish, and manage an MUH building partly or fully occupied by Hispanic residents in Los Angeles County. Bilingual doctoral students interviewed the managers and tape recorded their responses. Recordings ranged from 20 to 45 min in length. Managers received $20 in cash for their time and effort. The institutional review board approved the study in 2015. The interview guide was semistructured. A doctoral student transcribed the audio recordings verbatim.

The interview consisted of demographic characteristics, socioeconomic status variables, and closed-ended and open-ended questions about SHS/SHMS prevalence, knowledge, attitudes, behaviors, policies, resources, and educational preferences (Table 1).

**Table 1.** Open-Ended Questions of the Interview Guide

Interview questions
Knowledge and potential effects on people and property
1. Can you tell me some ways that SHS can enter an apartment?
2. Have you ever smelled SHS in the apartment building you manage/own? Would you consider SHS dangerous to your tenants' health such as causing asthma attacks?
3. Is there THS in your apartment building? Would you consider this dangerous to your tenants' health or bad for the building? Why?
4. Has a tenant ever complained about smoke (whether from tobacco or marijuana) in the building?
Policies
5. Do you know the policies in your city regarding smoking rules in MUH?
6. Do managers/landlords have the right to pass a smoke-free rule? How would you suggest to go about passing a rule and maintaining the building smoke free?
7. Do you believe a tenant should be required to move if he or she continues to smoke in an apartment unit or building when he or she has signed an agreement not to smoke?
Benefits
8. What good and bad things do you think would happen if you made your building smoke free?
Barriers
9. What are some concerns you currently have that make it hard for you to establish/maintain smoke-free rules in your apartment building?
Resources and suggestions
10. What are your suggestions on how to address exposure in MUH buildings?
11. Do you find the fotonovela a useful tool to communicate with tenants and key stakeholders about implementing/maintaining smoke-free rules? Why so?
12. What other resources would be useful to you?

MUH, multiunit housing; SHS, secondhand smoke; THS, thirdhand smoke.

### Data analyses

The transcripts were deidentified before analysis and combined with field notes. Data were cleaned and cleared of errors in Excel. Exploratory and descriptive analyses were conducted in Atlas.ti^®^. Narrative analysis of the open-ended responses was conducted by a bilingual and bicultural doctoral student. The coder considered meaningful statements those phrases that helped clarify the relationship between attitudes and behaviors regarding exposure to SHS/SHMS and existing policies. She coded meaningful statements and organized them for similarity, contrast, frequency, and causal and sequential order. Themes and frequency of codes were identified.

## Results

### Characteristics of the participants

Twenty managers of private housing properties agreed to participate in the interviews: 13 females and 7 males (Table 2). While recruiting managers, the data collectors found that some properties did not have on-site managers but were serviced by outside companies. Seventeen self-identified as Hispanic. All received payment for managing the building: partial or full waiver of rent for their unit, salary, or both. These caretakers managed an average of 50 units (min: 10 units–max: 225 units). The properties were located in 10 cities within Los Angeles County, representing a wide array of local policies and practices: Alhambra (2), Arcadia (2), City of Bell (1), El Monte (2), Glendale (2), Huntington Park (2), Los Angeles (2), Lynwood (3), South El Monte (2), and Whittier (2). Los Angeles County passed a tobacco smoking ban inside the units in publicly owned housing in 2013.^23^ At the time of data collection, only two of the cities surveyed, Glendale and Huntington Park, had passed tobacco/e-cigarette smoking bans in both public and private MUH.^24^

**Table 2.** Demographic and Socioeconomic Characteristics of the Managers (*N*=20)

Characteristics	Frequency (%)
Gender	
Female	13 (65)
Ethnicity	
Hispanic	17 (85)
Language	
English	6 (30)
Spanish	7 (35)
Bilingual (Spanish/other)	7 (35)
Country of birth	
United States	7 (35)
Mexico	5 (25)
Other country	8 (40)
Ethnic background	
European origin	2 (10)
Mexican	7 (35)
El Salvador	3 (15)
Guatemala	3 (15)
Other country/mixed ethnic	5 (25)
Age (min–max)	28–68 years old
Mean±SD	47.11±2.7
Education	
>HS	5 (25)
HS or GED	4 (20)
≥HS	11 (55)
Annual income (median)	$30k–$39k
Less than $10,000	3 (15)
$10,000–$19,000	4 (20)
$20,000–$29,000	2 (10)
$30,000–$39,000	2 (10)
$40,000–$49,000	2 (10)
$50,000–$59,000	3 (15)
$60,000–$69,000	2 (10)
$70,000 or more	2 (10)

GED, general education development; HS, high school; SD, standard deviation.

### Knowledge and potential effects on people and property

**FIG. 1.**
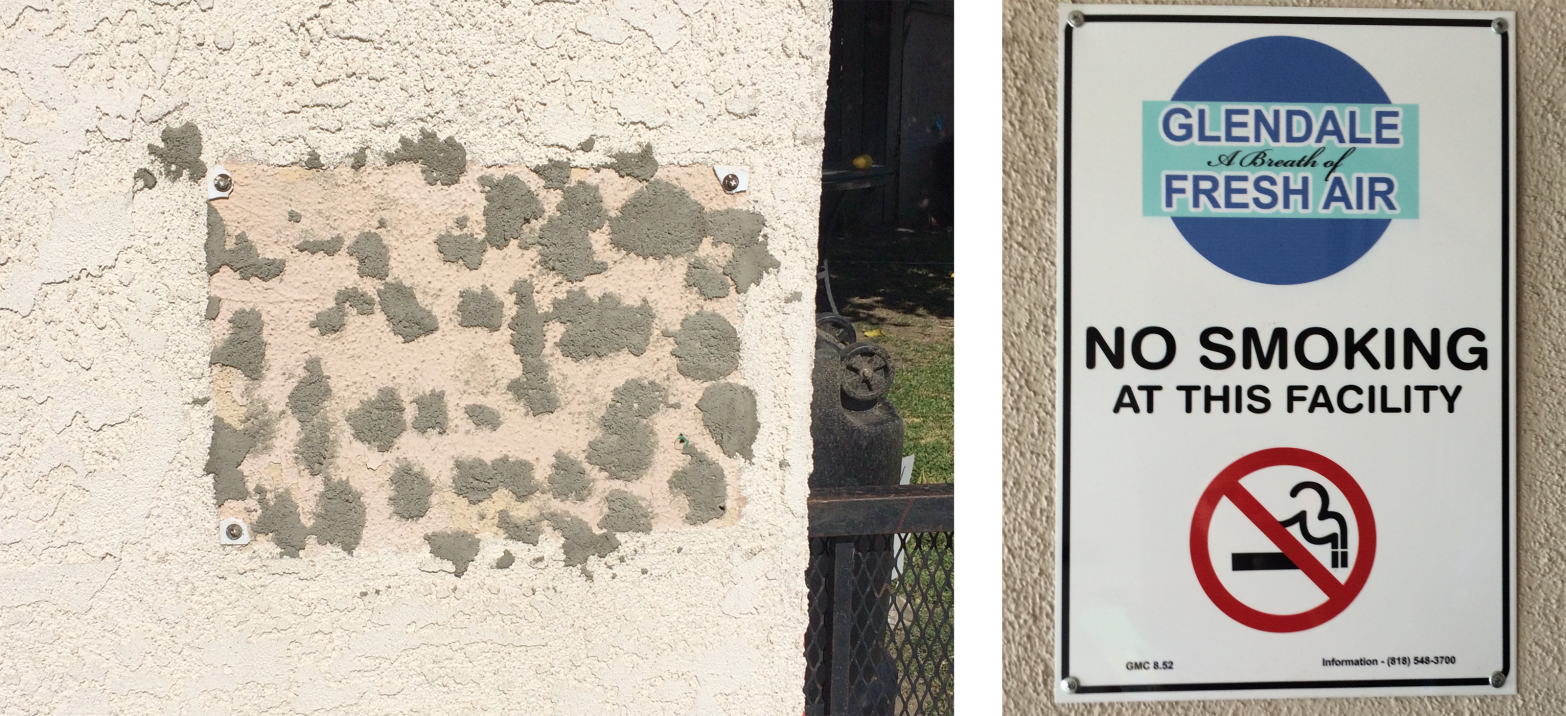
A generic no-smoking sign that was drilled to the wall was taken down by smokers (*left*). A no-parking/towing sign of similar size nearby was not removed. A no-smoking sign that was ratified by the City of Glendale's no-smoking law stood in place (*right*).

Only five managers had a smoker in their apartments, three were current or former smokers themselves (Table 3). The majority (18) were familiar with the term SHS, but only 1 manager was knowledgeable about THS and its effects. Although smoking *was not* allowed in outdoor areas in 14 of the cases, 19 managers had received a complaint about SHS, and 13 had received a complaint about SHMS (Table 3). Seventeen managers would probably or strongly favor a total ban on smoking on their buildings. While walking toward an interview site, the research staff noticed that in a couple of locations where tobacco smoking was banned, there was evidence of cigarettes within the prohibited zones (Fig. 1). Some participants understood the harm from being exposed to SHS:

“Yes, it hurts us more than 1st hand [smoke]. My daughter and another tenant have emphysema, a couple of tenants have asthma. Yes, I have smelled SHS, when they smoke around here, tobacco or marijuana, we can smell it.” (Mexican woman, non-smoker age 60)

“SHS is the product that is emitted from a smoking cigarette or any type of product that is like cigarettes after they smoke it, we ingest it. It's absolutely bad for you… My husband has asthma and he smokes. SHS can trigger asthma attacks, even for people who don't have chronic respiratory illnesses you'll see that if we're around SHS we'll find ourselves coughing and everything else. (Mexican Spanish woman, non-smoker age 42)

“If people know what you're cooking, they can smell your smoke.”

(Mexican woman, non-smoker age 50)

While most managers were sympathetic to the tenants' rights to fresh air, a couple of managers saw the issue from the smokers' perspective:

“If you don't want to smell the smoke, then close the windows. People have a right to smoke, and people have freedoms in this country.” (Romanian woman, non-smoker age 61)

**Table 3.** Characteristics of the Managers' Living Conditions, Rules, and Behaviors Regarding Multiunit Housing Tobacco and Marijuana Smoking Rules

Managers' living conditions and rules (*N*=20)	Overall, *n* (%)
Living conditions	
Lives with a person who uses tobacco products	5 (25)
Who is the person who uses tobacco in their homes	
Self	3 (15)
Their spouse/partner	1 (5)
Their child	1 (5)
Lives with a person who has a chronic heart or lung condition	8 (40)
A tenant has complained about tobacco smoke	19 (95)
A tenant has complained about marijuana smoke	13 (65)
A tenant has complained about smoke from vape	1 (5)
A tenant has smoked marijuana for medicinal reasons	4 (20)
Knowledge and beliefs	
Knows the definition of the term secondhand smoking	18 (90)
Beliefs SHS is harmful to tenants' health	20 (100)
They have experienced SHS in their building	17 (85)
Knows the definition of the term thirdhand smoking	1 (5)
Beliefs THS is harmful to tenants' health	19 (95)
Beliefs THS is harmful to the building	16 (80)
They have experienced THS in their building	14 (70)
Knows the city ordinance on SHS in MUH	6 (30)
Beliefs they can enact rules on their own	7 (35)
Rules and preferences	
Manager allows smoking inside their homes	0 (0)
Smoking is allowed in outdoor common areas	6 (30)
Smoking is allowed in indoor common areas such as hallways and laundry rooms	7 (35)
Would favor a rule in their building that bans smoking in all areas	13 (65)
Would favor separate smoking and no smoking areas	7 (35)
Would favor a rule banning smoking medicinal cannabis	12 (60)
Would favor evicting a tenant who breaks the rule	20 (100)
Would favor contact information of government agency for help (*n*=19)	13 (69)
Thinks the fotonovela is a good educational strategy for tenants (*n*=19)	16 (84)

The managers were unanimously in agreement about the dangers of SHS to the residents' health, and while they were not familiar with the term THS, once explained most of them could assert that THS can be harmful to the tenants and the building.

“We had an older gentleman living in the 3rd floor, heavy smoker. We literally had to cut out the whole apartment, throw everything away. I mean, carpet, lining, they must've put 5 or 6 coats of paint, but before that they must've put a special chemical to get some of the nicotine off the wall.” (Chilean man, non-smoker age 47)

### Policies

Although only six managers felt that they were informed about their city's policies on smoking on MUH; only two of them were correctly informed of the existing policies for MUH in their respective cities.

“I don't know what restrictions exist. I work for the owner, he's never told me about rules, I haven't brought it up to him. We've never spoken about that…I would have to talk with a lawyer to know if I can [pass rules], before enacting any rules. I need to know if I'm breaking anybody's rights. Law counseling would be helpful”

(Guatemalan man, smoker age 47)

“To be fair I would favor a rule to have 2 areas, a smoking area… I could create a smoking area by the barbecue area but there wouldn't be anyone to enforce it 24/7.” (European American man, smoker age 68)

Twelve participants would prefer to ban smoking marijuana for medicinal reasons; the rest would respect patients' rights and evaluate making an exemption on a case by case basis. Over half of the participants consider smoking marijuana as dangerous as tobacco or even worse, about one-third view marijuana as healthier than tobacco because of the continuous media coverage presenting all modalities of marijuana as having only healing properties.

“Same as with tobacco, bad to business and tenants' health, except it brings a bad reputation to the business. As for marijuana, the rule is no smoking period, regardless if it's recreational or medicinal, even if they have a card, ‘cause they can say I have a card, then do it outside the property. Until we get a city official where it's mandatory for landlords to allow a certain type of smoking, it's completely off limits.” (Mexican American man, non-smoker age 31)

“Personally, I'm opposed to allowing the use of medicinal marijuana because there are other methods to heal pain.” (Salvadorian woman, non-smoker age 41)

“I don't think that SHMS is as bad as tobacco. I cannot explain to you why, but marijuana is a plant, so I don't think it's as bad as tobacco.” (Mexican woman, non-smoker age 50)

Managers expressed ambivalence about the government policies and the internal rules of their companies with regard to marijuana smoking:

“For medicinal marijuana use is a different thing, people that go down and get a doctor permit for medicinal marijuana that's ok, it's like medicine. But I don't think that I would appreciate having that happen in my building. I haven't come across that scenario so I would have to think about how to handle it.” (European American man, smoker age 68)

“No, because all the rules that I give must have been laws from the city first or municipal code of Los Angeles. I have to base my rules on the laws of the city, I cannot make rules that go against the current [marijuana] laws.” (Mexican woman, non-smoker age 40)

The overwhelming majority of the managers felt that the landlord or company would have the ultimate say, unless the city enacts a law first. Some managers are afraid to be assertive about enforcing the rules. All managers unanimously concurred that tenants who repeatedly break the rules agreed upon in their lease contracts must be evicted. Seventy percent would like to have the contact information of a government agency that can help them address issues related to SHS/SHMS.

### Benefits

The top 3 benefits of banning smoking reported were as follows: (1) most tenants prefer to live in smoke-free apartments (14), (2) it costs less to clean the apartment when a tenant moves out (10), and (3) the owner can rent the units for more money (8).

“It doesn't smell like an ashtray. Smell is better, no cigarette butts, I don't have to worry about kids picking up butts.” (Mexican and Spanish woman, non-smoker age 42)

### Barriers

The most common barriers to preventing SHS/SHMS cited by the managers were as follows: (1) There is no place at least 25 feet away from the premises to designate as a smoking area (14), (2) cultural differences between the tenants (8), and (3) legal problems/lack of legal language to prepare documents (6). The feedback reflected the diversity of Los Angeles County. Hispanic residents are scattered throughout the county. MUH complexes in the geographic locations assessed have various concentrations of Chinese, Koreans, Armenians, Russians, black/African Americans, and European Americans. To the respondents it is pertinent that everyone gets educational and legal information in their languages (i.e., contracts, eviction notices, and signs).

“There's one person who speaks Spanish and doesn't speak English, but others translate.” (Mexican American Man, smoker age 58)

“The sign was in Chinese because the majority of smokers in this building are Asian, but I let them smoke there even though they still leave cigarettes butts around the garbage bin, they don't even put it inside the bin. You can see it in the back when you come out. A girl who speaks Chinese helps me translate the signs, it's been raining so the sign fell.” (Mexican woman, non-smoker age 50)

Despite encountering long-term exposure to environmental smoke, Hispanic residents and immigrants do not move out as they have the dilemma of finding affordable housing. Therefore, filling units is not a problem for the managers despite raising rental fees and odor from smoke. In the open-ended responses, enforceability was the biggest barrier that managers face:

“We have it in the contract, but people they don't follow rules. Anything about smoking but people don't listen to us. It's not allowed anywhere. I would prefer no smoking anywhere. I put no smoking signs, but they took them down already (Figure 1). My little sister used to have asthma… I think smelling smoke is bad for her. I'm pregnant, SHS affects my baby too.” (Salvadorian woman, non-smoker age 28)

“The tenants could not sue us if it was a law, they'd have to go through the city first.” (Chilean man, non-smoker age 47)

In essence, managers feel that when they are kind and respectful of the smoker tenants, smokers are responsive to their requests. However, eventually smokers revert due to a lack of knowledge of smoking areas in their vicinity. Enforcement is difficult in the long term as the managers do not have authority over outside smoker neighbors. For those managers whose residents followed the nonsmoking rules, often guests and intruders complicate compliance and enforcement. In some instances, teenagers sneak into the properties to smoke substances without permission. Respondents think that allowing police officers to ticket smokers for infringing no-smoking zones might deter them from relapsing or trespassing.

### Resources and suggestions

The majority of the participants loved the idea of using the fotonovela *El Reto de Marta-Marta on a Mission* to educate their tenants (16). They liked the story line and personally identified with the scenarios and characters. They seem delighted while leafing through it, smiled, nodded, and laughed.

“[I like this], especially for Spanish speakers, for this building. Like the first scene where the wife passes by the 2 guys that are smoking outside, that happens here a lot.” (Pacific Islander man, non-smoker age 36)

They would appreciate the following resources. (1) Contact information of local government agencies that can assist them to enforce the rules (14), (2) signage for no-smoking and smoking areas (11), and (3) lease language (8). Participants had the following suggestions. Involving the government as much as possible, designating public smoking areas in neighborhoods away from living quarters and children's play areas, passing laws, educating tenants and managers about current smoking laws, specifying smoking laws in the contracts clearly, putting up universal no-smoking and smoking signs, distributing a map of locations where people can smoke, requiring managers to attend training by agencies such as the U.S. Department of Housing and Urban Development, and having the police enforce the new laws with fines.

“We bought signs but we're gonna put new ones. 12” by 18” Signs are $4.99, it wasn't very expensive and the actual plaque that goes on the door was $8. The universal sign is very clear for anybody, Chinese speakers and everybody. Even with broken English it's easy to communicate with a smoker that there's no smoking here. [She signaled with her hands no smoking and pointed towards the door]. Most of the time they say ok, ok, no problem.” (Mexican and Spanish woman, non-smoker age 42)

“A government policy to prohibit smoking in apartment buildings. It would be best if the government sponsored a no-smoking policy for us.” (Salvadorian man, non-smoker age 48)

“Our company makes us go to seminars every 3 months and we have to cover all of the scenarios and that's one of them [THS]. Our company puts them on, they just hire experts and smokers and have people from attorney's offices to talk about legal troubles, and people from Fair Housing they give us seminars.” (Chilean man, non-smoker age 47)

## Discussion

Managers are aware that most tenants prefer to live in smoke-free properties.^25,26^ Respondents did not believe that they were able to pass rules without the backing of the landlord or management company. In accordance with their beliefs, by law, in California, landlords have a right to enact smoking policies, however, as shown in this study, some may not. Government assistance in passing and enforcing laws could deter smokers from breaking property rules.

As of 2016, California has passed laws equalizing e-cigarettes with tobacco cigarettes, yet no law has been proposed to protect MUH tenants from exposure to marijuana smoke. New items such as water filtration vaporizers minimize the smell of SHS/SHMS to outsiders, but over time the aerosol may still penetrate surfaces and be difficult to remove from inside of the units when the tenant moves out.

The majority of the managers expressed concern with allowing their tenants to smoke marijuana for medicinal or recreational purposes due to the undesirable adverse effects, penetrating smell, and exposure to the surrounding children. Delta-9-tetrahydrocannabinol (THC) is the major active ingredient of marijuana.^16^ Depending on the patient's condition, THC and other active ingredients can be administered in nonsmoking modalities such as oral tablets, capsules, sublingual sprays, intramuscular injections, or edibles.^27^ These marijuana-based medications could be assessed for consumer use in the United States to lessen the burden on environmental pollution. With regard to smoking recreational marijuana, the preference was to prohibit it as well as any other smokable products (i.e., e-cigarettes, hookah, and vape). Exceptions could be made in a few cases where there is a medical diagnosis.

Ethnically diverse tenants encountered cultural and linguistic barriers to address unwanted exposure to environmental smoke. Hispanics favor saving face and not confronting people directly, unless they have established a relationship with this person.^10^ While low-income Hispanics are disproportionally affected by SHS, THS, and SHMS, they also face limited mobility opportunities. Guests and intruders complicate the matter. The managers believe that a law banning smoking of all recreational substances in MUH can help them enforce house rules and avoid interpersonal conflicts among residents.

On-site managers make presence in the properties, represent the community, know the residents, and care about their families. Unfortunately, landlords are outsourcing these valuable jobs to off-site companies. In such a situation, the cities could require companies conduct periodic inspections of the units and assess residents' feedback on compliance. Our findings confirm previous research that highlights the need to educate stakeholders about the contents and burden of THS.^28^ Being able to inspect units might help prevent the accumulation of THS over time.

Health communication guidelines recommend producing educational materials at sixth grade reading level or lower. As discussed by our participants, the proposed fotonovela would be understood by Hispanics and could be well received by other ethnic groups. Managers identified key points for dissemination of information: when a new law/house rule is passed, when a person signs a new lease, when residents pay their rent, or during manager/tenant meetings. Disseminating a map of smoking areas in the neighborhood and universal smoking/no-smoking signs along with the fotonovela may increase adherence to the rules.

Most MUH managers report receiving complaints of tobacco and recreational marijuana smoke infiltration. Results of this qualitative study revealed that apartment managers unanimously support various forms of smoking policies that include tobacco, marijuana, and any other smokable products that are invented in the future. Most managers report low agency in being able to pass and enforce no-smoking rules. Therefore, new government policies, manager trainings, tenant education, and ways to enforce rules are necessary to protect low-income apartment tenants from SHS and SHMS. Such laws may overcome interpersonal conflicts arising from cultural differences and ambiguity of house rules. Educational strategies should coincide with the timing of key manager/tenant events. Findings back our previous results from a survey of Hispanic tenants and can be used in policy development and educational interventions to lower MUH residents' exposure to SHS/SHMS.

### Limitations

The findings of this qualitative study are limited by the number of participants. While the sample might not be representative of all situations across Los Angeles County, the buildings were located in 10 cities over Los Angeles County, representing a wide array of local policies and practices. A sample of 20 interviewees was enough to reach saturation of responses for qualitative research. The data were cross-sectional, and we did not assess for knowledge changes after exposure to the educational material.

## Abbreviations Used

HUD: Housing and Urban Development
MUH: multiunit housing
SHMS: secondhand marijuana smoke
SHS: secondhand smoke
THC: delta-9-tetrahydrocannabinol
THS: thirdhand smoke
